# Juvenile idiopathic arthritis in a child with nijmegen breakage syndrome

**DOI:** 10.1186/1546-0096-12-S1-P204

**Published:** 2014-09-17

**Authors:** Agnieszka Gazda, Beata Kołodziejczyk, Małgorzata Pac, Lidia Rutkowska-Sak, Iwona Czerwińska-Kartowicz, Ewa Bernatowska

**Affiliations:** 1 Clinic of Paediatric Rheumatology, Institute of Rheumatology, Warsaw, Poland; 2 Department of Immunology, The Children's Memorial Health Institute, Warsaw, Poland; 3 Institute of Mother and Child, Warsaw, Poland

## Introduction

Nijmegen Breakage Syndrome (NBS) is a rare autosomal recessive DNA repair disorder, caused by mutation in the NBS1 gene on chromosome 8q21. The hallmark symptom is microcephaly, other characteristic features are facial phenotype, growth retardation, premature ovarian failure in girls. Psychomotor development is usually not disturbed. Impaired cellular and humoral immunity is essential feature of syndrome. Patients with NBS have an extremely high risk for developing malignancy.

## Objectives

We present 7 y.o. girl with NBS who developed arthritis.

## Methods

The child was delivered in time, by cesarean section due to IUGR, with congenital defects syndrome (microcephaly, dysmorfic face, anal atresia, fistula to the skin of the perineum). She underwent surgery shortly after birth. Genetic consultation confirmed, c.657del5 mutation on both alleles of *NBS1* gene, normal karyotype and aberration of chromosome7 and 14. In first few years she suffered from upper respiratory tract infection, viral gastrointestinal infections. At the age of 5 years replacement IVIG was introduced. Since 3^rd^ year of life the granuloma– like skin changes on ears, nose and hands had been observed (epithelioid granuloma in histopatological examination). The immunological assessment, done on IVIG showed slightly diminished level of IgG, normal IgA, and slightly increased IgM. Immunophenotyping of lymphocytes revealed severely diminished number and percentage of CD3+/CD45+ T cells (27.3%), CD3+CD8+/CD45+ cells (5.3%), CD4+CD3+/CD45+ cells (15.8 %), with B cells and NK cells within the normal range. The *in vitro* response of lymphocytes to mitogenic stimuli such as phytohaemagglutinin, anty-CD3 and Pansorbin was deeply decreased. Slight reduction of lymphocytes with no leucopenia was observed.

## Results

Since 3^rd^ year of life inflammatory process in joints was obserwed: the swelling, effusion of right knee, swelling of right wrist and 3rd and 4th finger of the right hand. The X-ray of the right knee was correct. In ultrasound inflammation was confirmed in the knee, tendovaginitis of the extensor of 4th finger tendon and flexor of 3rd finger of the right hand. ESR and CRP were in norm, trombocytosis 508G/L, Rheumatoid Factor-352 IU/ml, Anti CCP antibodies (-), antigen HLA B27(+), inflammatory character of synovial fluid, sterile join fluid cultures. The patient received i.a. glukokortykosteroid (GKs), IVIG 1mg/kg/month with improvement. In 2013 after pharyngitis exacerbation of arthritis: swelling and effusion in the knee, enlargement of the knee which suggested hypertrophy of epiphysis the bones of the right knee, swelling and limitation of motion in the right wrist, swelling of 3rd, 4th finger of the right hand and 2nd,3rd finger of the left hand with tendency to flexion contractions in proximal interphalangeal joints. In ultrasound–chronic active inflammatory process in knee, tendinitis and tendovaginitis in hands. In treatment the i.a. GKs and NSAID were used, then methotrexate.

## Conclusion

The clinical symptoms and USG confirm diagnosis of juvenile idiopathic arthritis in patients with NBS. Arthritis, presence of HLA B27 antigen but also presence of RF authorize to diagnosis the JIA -onset form other arthritis.

## Disclosure of interest

None declared.

